# Spatio-chromatic vision with multifocal diffractive intraocular lens

**DOI:** 10.1186/s40662-023-00350-5

**Published:** 2023-08-01

**Authors:** Maria S. Millan, Laura Clavé, Aurora Torrents, Jesús Armengol, Fidel Vega

**Affiliations:** 1 Applied Optics and Image Processing Research Group, Universitat Politècnica de Catalunya-BarcelonaTech, C/ Violinista Vellsolà, 37, Terrassa, 08222 Barcelona, Spain; 2 Mataró Hospital, Consorci Sanitari del Maresme, Barcelona, Spain

**Keywords:** Presbyopia-correcting intraocular lens, Spatio-chromatic vision, Multifocal intraocular lens, Diffractive lens, Visual acuity, Energy efficiency, Modulation transfer function, Longitudinal chromatic aberration

## Abstract

**Background:**

This study aims to detect alterations in the spatio-chromatic pseudophakic vision produced by multifocal diffractive intraocular lenses (IOLs) and provides a physical interpretation.

**Methods:**

In vitro characterization of the imaging performance of two diffractive IOLs: AT LISA Tri (Zeiss) and FineVision (PhysIOL) in on-bench model eye illuminated with red (R, 625 nm), green (G, 530 nm) and blue (B, 455 nm) lights. We used the metrics: energy efficiency (EE), area under the modulation transfer function, longitudinal chromatic aberration (LCA), and halo intensity. Through-focus (TF) analysis and calculation of the expected defocus curve under white (W) daylight were included. In vivo visual acuity (VA) of 50 pseudophakics (60 eyes) was assessed under W, R, G, B lights at far and near. Two clinical experiments evaluated LCA and R, G, B TF-EE effects on pseudophakic vision and their relative importance.

**Results:**

Clinical mean VA values under W light agreed with the predicted values at far and near for both IOLs. LCA measurements and R, G, B TF-EE curves were consistent with their lens design based on the 0th and 1st diffraction orders operative for far and near vision, respectively. LCA effects were compensated at near but noticed at far (− 0.75 D under B light). We detected strong asymmetry in visual resolution depending on the object distance and the illuminating wavelength—red predominance at far, blue predominance at near—in consistency with the TF-EE measurements.

**Conclusions:**

Diffractive multifocal IOL designs produce asymmetries in the spatio-chromatic vision of pseudophakics beyond the alterations strictly due to LCA. VA asymmetry for far/near object distance under R and B illumination is clinically detectable in subjects implanted with IOLs with 0th and 1st diffraction orders for far and near vision, respectively. Such VA asymmetry cannot be explained solely from the influence of defocus, as would be derived from a chromatic difference of power, but mainly from the wavelength dependence of the EE.

**Supplementary Information:**

The online version contains supplementary material available at 10.1186/s40662-023-00350-5.

## Background

Modern cataract surgery with intraocular lens (IOL) implantation can restore human vision far beyond the degradation produced by the loss of transparency of the natural lens. Significant improvements have been introduced in IOL designs to compensate for common refractive errors (such as myopia, hyperopia, astigmatism), age-related insufficiencies (such as loss of accommodation or presbyopia), and some image degradations (such as high-order and chromatic aberrations). Remarkable scientific and technological advances in eye modelling, ray-tracing calculation, in vivo biometry, adaptive optics and laboratory testing on optical bench, along with intensive clinical research, have led to define a number of optical metrics that correlate with postoperative outcomes and can even be used to predict the visual quality of the average patient after surgery [1–3]. This may help surgeons to make a more informed decision about the IOL to choose in a scenario of over one hundred designs and products. Moreover, patients can even experience prospective vision before undergoing surgery by means of visual simulators with active elements able to display the optical function of a given IOL [4].

Although optical bench testing and computer eye model simulations are very useful for understanding IOL performance in a range of observation distance, there is a gap between physics and perception that requires investigation for further insight. Thus, for example, while quite a few studies, data, and calculations are carried out under monochromatic light (typically in the green spectral region corresponding to the maximum photopic efficiency), human vision is mostly realized under white light. In this field, considerable attention has been paid to the longitudinal chromatic aberration (LCA), caused by the dispersive nature of materials, which produces a variation of the refractive optical power with wavelength. Even though human vision is highly tolerant to LCA in the presence of natural monochromatic aberrations (about 2.1 D of chromatic difference of refraction in the visible spectral range from 400 to 700 nm) [5], LCA has gained new interest because it can be manipulated through IOL design, in particular, with the introduction of hybrid refractive-diffractive IOLs (hereafter, for the sake of brevity, referred to as diffractive IOLs). This type of IOLs is based on engraving a diffractive profile on at least one of the surfaces of a refractive lens, which is used as a carrier platform. The hybrid component is specifically designed to provide coaxial multifocality, and therefore, distinct vision for several foci. The focus of lower optical power allows for distance vision, while the focus of higher optical power allows for near vision. Since a multifocal IOL forms simultaneous images of the same object, subsequent neural adaptation is necessary to allow the subject to focus on the image of interest despite being overlaid by at least one out-of-focus image [6]. In any case, it involves contrast reduction and the potential appearance of perceptual dysphotopsia.

Diffractive bifocals provide enhanced vision for far and near distances whereas trifocals are intended to further improve vision at intermediate distance. Depending on the diffraction orders involved in its multifocality, the diffractive component can mitigate the LCA produced by the dispersive nature of the IOL material and the ocular media [7, 8]. The joint compensation of LCA and corneal spherical aberration (SA) is advantageous for contrast sensitivity enhancement [9] and has been used to design a diffractive bifocal IOL of low addition for extended range of vision [10]. LCA in pseudophakic eyes has been intensively evaluated [11–14] and measured [15–18], but has barely gained enough clinical relevance, possibly because of the natural attenuation of LCA in normal human vision [5] and the increasing use of IOL materials with relatively low chromatic dispersion (e.g., the pseudophakic chromatic difference of refraction with an IOL Abbe number of 47 is similar to that of normal human eyes [11] and IOLs with even larger Abbe number are currently available).

Another perspective of the issue leads us to consider the fraction of the incident energy deviated to each focus. The energy efficiency (EE) metrics accounts for the distribution of energy between the different foci and is typically featured for the design wavelength (546 ± 10 nm as recommended by the International Standard Organization ISO 11979-2:2014) [19]. In diffractive components, both the optical power and the EE depend strongly on the wavelength. Since the multiple foci are coaxial, their positions, relative peak intensities, contrast, and blur turn out to be physically dependent on the wavelength. As a result, the optical image quality—evaluated through modulation transfer function (MTF)-based metrics—becomes wavelength dependent as well. Labuz et al. [20] detected the effects of a 580 nm high-pass red filter on the visual acuity (VA) and the contrast sensitivity of patients implanted with a low add diffractive IOL. In comparison with white light viewing, the red filter did not improve far vision but had an adverse effect at the near and intermediate distances. Their MTF measurements showed that the diffractive IOL was “intermediately dominant in the blue light but far dominant in the red light”. The result reported by Labuz et al. [20] was, in fact, initial evidence to further motivate our study.

The effects of the optical power and EE wavelength dependency showed by multifocal diffractive IOLs on the visual quality of pseudophakic subjects are still an open issue. In this cross-sectional study with laboratory investigation, we searched for the possible spatio-chromatic changes with the observation distance that pseudophakic vision may experience because of a physical fact: the wavelength dependency of the multiple images formed by a diffractive multifocal IOL. Should there be noticeable changes, their evaluation and potential consequences would contribute new knowledge to vision science. In addition, the understanding, interpretation, and description of their effects in terms of the optical features of the diffractive lens would be of practical interest for IOL designers and clinical practitioners.

We show the impact of the spectral dependency of diffractive IOLs on the spatial and chromatic vision of pseudophakic eyes and give a physical rationale based on the optical design of the lens. We assess the effects of the wavelength dependency of EE and compare them with the effects of LCA for different observation distances using both in vitro and in vivo methods. To this end, we characterize IOL performance using well established laboratory techniques based on a model eye on optical bench (optical experiment) [7]. A VA expectancy in a variety of observation conditions will be computed from the optical quality results and compared with the actual VA outcomes of pseudophakic patients in similar conditions. This study aims to assess the joint effects of LCA and EE on pseudophakic vision (clinical experiment 1) and their relative importance. To achieve the latter, LCA will be compensated in a second clinical examination (clinical experiment 2) and the effects of the EE alone will be evaluated and discussed.

## Methods

### Optical setup

The test bench with the model eye used to measure the optical performance of the IOLs in vitro is shown in Additional file 1: Fig. S1. The setup, described in detail elsewhere [3, 21, 22], consists of three parts: the illumination system, the model eye, and the image acquisition system. A light emitting diode (LED) illuminated a test object placed at the front focal plane of a collimator (200 mm focal length) to locate the object optically at infinity from the model eye. We used a set of three red (R), green (G), and blue (B) LED sources, with various spectral band emissions (Additional file 1: Fig. S2 and Table S1) and two object tests (Additional file 1: Fig. S1): a pinhole (200 μm) for EE and halo measurements, and a four-slit pattern for MTF measurements. The slits were 10 μm wide. The model eye, formed by an artificial cornea lens and a wet cell where the IOL was immersed, met ISO 11979-2:2014 (model eye type 2) recommendations [19]. The cornea lens was an achromatic doublet (Lambda-X, Belgium) intended for the evaluation of aspheric IOLs; it induced SA =  + 0.16 μm (in terms of the Zernike c[4,0] coefficient) for a 5.15 mm pupil at the IOL plane. An iris diaphragm placed in front of the artificial cornea controlled the lens aperture. The pupil diameters mentioned in this work are referred to the IOL plane (hereafter named IOL pupil). The image acquisition system was composed of a 10×, infinity-corrected, plan-achromatic, microscope objective assembled to an 8-bit CCD camera, mounted on a high precision, three-axis translation holder for through-focus (TF) analysis. The image acquisition unit (microscope and camera) was nearly diffraction-limited across the visible spectrum with a cut-off frequency of 555 cycles/mm. To reduce the impact of electronic noise, each image was the result of temporal averaging eight frames at a time.

### Metrics

The basic metrics used for the optical characterization of the IOLs were the EE, the area under the modulation transfer function (MTFa) and the halo size and intensity. We considered two IOL pupils of size 3.0 mm and 4.5 mm. EE and MTFa were measured under separate R, G and B illumination, within a TF span of image vergence ranging from − 4 D to + 3 D, in 0.10 D steps. The origin of image vergence and defocus (0.0 D) was set at the distance image (highest MTF value at 50 cycles/mm) for the G light (530 nm, close to the standard design wavelength of 546 nm) [19]. The spatial frequency 50 cycles/mm corresponds, in an eye of 17 mm focal length, to 15 cycles**/**degree in the object space. Negative dioptric value corresponds to near vision vergence according to the clinical convention.

The EE was computed through the light-in-the bucket [23] measurement of the pinhole image formed by the model eye (Additional file 1: Fig. S3a). Basically, the image core energy (E_core_) to the total energy (E_total_ = E_core_ + E_background_) ratio approaches the light-in-the bucket value in the experimental practice [7]. LCA equalled the refractive power difference calculated from the distance between the R and B EE peaks in the image space. Positive sign was assigned to LCA when the power magnitude for the B light was higher than that for the R light, and the negative sign was assigned the converse.

The MTF for a given image vergence within the TF range (Additional file 1: Fig. S3b) was computed from the image of the four-slit test formed by the model eye with the IOL immersed, as reported elsewhere [2]. The MTFa was calculated by integrating the MTF curve in the spatial frequency range from 0 to 50 cycles/mm. The TF-MTFa data have been used to calculate the expected VA and depth-of-focus as measured in clinics through postoperative defocus curves [1, 2, 24].

For the sake of a closer prediction to the postoperative VA (logMAR) defocus curves, we considered the chromatic characteristics of the W LED used in the clinical experiments of this study. From the chromatic coordinates of the R, G, B LEDs in the CIE 1931 system {R(0.7017, 0.2981), G(0.1224, 0.7478), B(0.1506, 0.0262)} (Additional file 1: Fig. S2 and Table S1), we calculated the R:G:B power ratio that would generate the W light of chromatic coordinates (0.3128, 0.3292) (6500 K daylight). We used only the chromatic data of the LEDs, not their relative intensities, because the R, G, B image channels were normalized separately prior to the MTF calculation i.e., adjusted to cover the grey level dynamic range of the camera sensor with no saturation. Following the procedure described by Hooi [25] and Huang et al. [26] we determined the R:G:B power ratio 4:10:1. This ratio provided the weight coefficients to calculate the linear combinations of the polychromatic metrics EE_poly_ and MTFa_poly_ in the TF range and, hence, to compute the expected VA and postoperative depth-of-focus, which are figures of clinical interest [1–3, 24]. To this end, we used the mathematical expression$VA=A\mathrm{exp}\left\{B*{MTFa}_{poly}\right\}+C$, with$A=1.828 , B=-0.23 , {\text{and}} C=0.014$, which was found to reach the correlation coefficient ${R}^{2}=0.94$ in a former work [3].

Halo was characterized from the image of the pinhole test, in the far and near foci of each IOL, under the R, G, B lights. Logarithmic scale of intensity was used for the sake of halo visualization (Additional file 1: Fig. S4).

### Intraocular lenses (IOLs)

We used two off-the-shelf 20 D trifocal IOLs: AT LISA tri 839 MP (Carl Zeiss Meditec AG, Jena, Germany) and FineVision Micro F (PhysIOL S.A., Liège, Belgium) for the in vitro on-bench optical testing. Both IOLs are made of hydrophilic acrylic materials with 1.46 refractive index and 58 Abbe number. They are refraction-based in far vision, meaning they use the 0th diffraction order, which has no associated power. They have a combination of two diffractive profiles, of different add power, that use their 1st diffraction orders for intermediate and near vision. Both designs are pupil dependent since their diffractive profiles have different heights across the aperture:

- FineVision: apodized combined diffractive profiles 1 (+ 1.75 D) and 2 (+ 3.50 D) on the entire optic surface (6 mm diameter). Active diffractive orders: Far (0th order of the two profiles), intermediate (1st order of 1st profile), near (1st order of 2nd profile and, with little contribution, 2nd order of 1st profile). Posterior aspheric surface. The lens induces SA (c[4,0] =  − 0.11 µm for 6 mm entrance pupil).
- AT LISA tri: two zones in the aperture. Central zone (4.34 mm): combined diffractive profiles 1 (+ 1.66 D) and 2 (+ 3.33 D) for trifocal imaging. Periphery (until 6 mm): bifocal, far and near imaging. Active diffractive orders: Far (0th order), intermediate (1st order of 1st profile), near (1st order of 2nd profile). The lens induces SA (c[4,0] =  − 0.18 µm for 6 mm entrance pupil).

These trifocal IOL designs have been intensively studied in related research. The interested reader will find further information elsewhere [22, 27–29].

### Clinical data

**Fig. 1:**
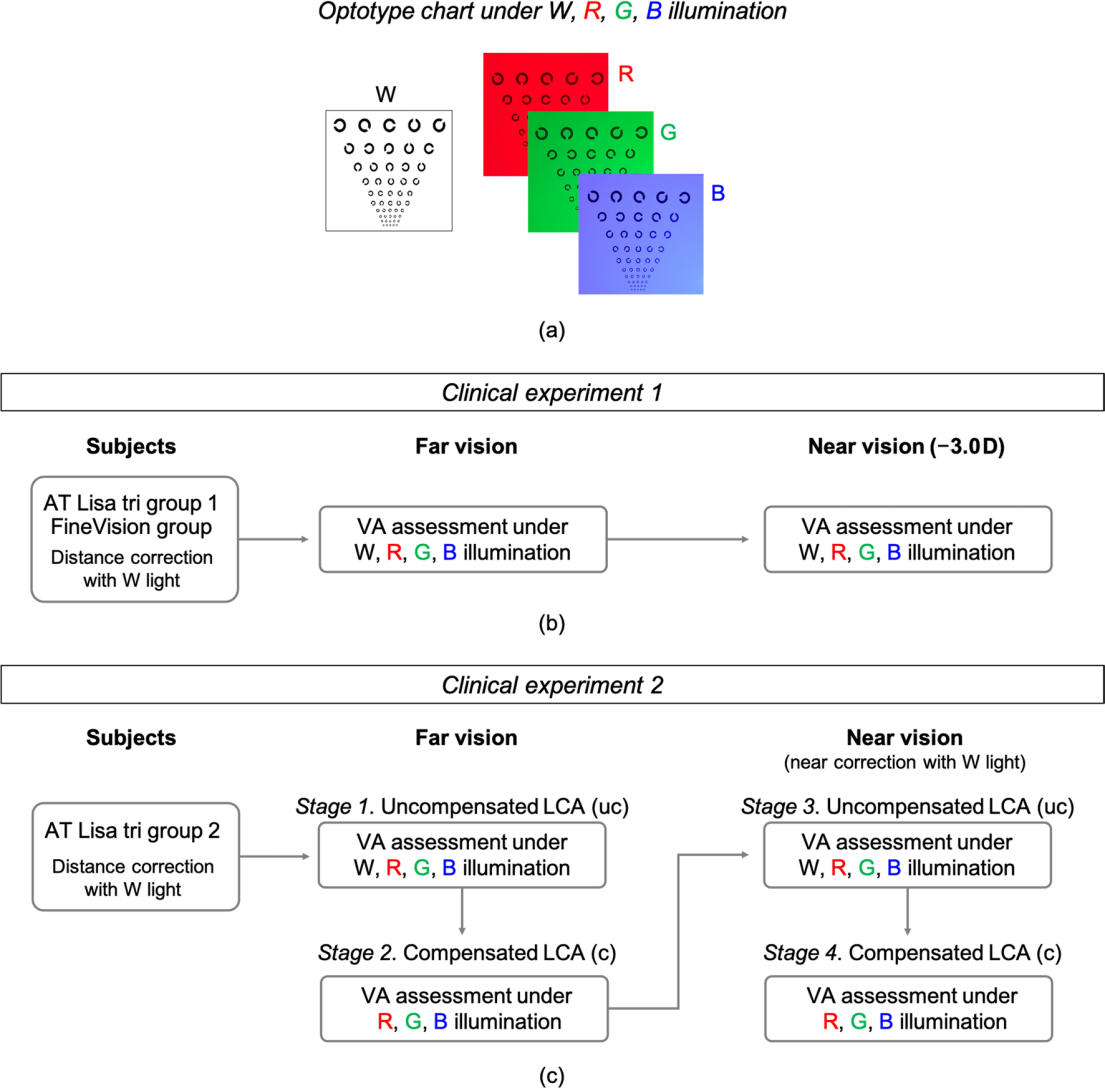
Visual acuity assessment. **a** Sequential W, R, G, B illumination of the optotype chart; **b** Flowchart for clinical experiment 1; **c** Flowchart for clinical experiment 2. VA, visual acuity; W, white; R, red; G, green; B, blue

Fifty patients (60 eyes), aged 49 to 74 years, were classified into three groups (Table 1): FineVision group, AT LISA tri groups 1 and 2. Clinical experiment 1 was followed by the FineVision group and the AT LISA tri group 1, whereas clinical experiment 2 by the AT LISA tri group 2 (Fig. 1). These clinical experiments constituted a cross-sectional study which followed the tenets of the declaration of Helsinki. Subjects were fully informed about the study and provided written consent. The ethics committees of the Hospital de Mataró (Consorci Sanitari del Maresme, Barcelona, Spain) and other collaborative centers approved the clinical study (CEIm 20/19 LIO2019). All examinations were carried out by a single experienced optometrist (LC) using the same material and procedures. Eligible patients presented bilateral cataracts and no comorbidities. They underwent symmetrical bilateral cataract surgery, meaning they were implanted with the same type of lens, using similar technique—phacoemulsification followed by IOL implantation into the capsular bag in both eyes. Eye retinoscopy and subjective refractions were performed for all patients. Specific inclusion criteria were preoperative refraction error (spherical equivalent) less than ± 5.0 D, postoperative best distance corrected VA better than 0.1 logMAR, availability and willingness to comply with the examination procedures. Since the measurements were not conventional in both experiments, dedication and collaboration were requested from the recruited subjects. Key exclusion criteria were complications during or post-surgery, abnormalities in colour vision, prior ocular pathology, or ocular surgery, including refractive procedures. The examination was done between one and six months after surgery. The chart was placed at 3.5 m for far VA assessment, so the object vergence of − 0.25 D was included in the manifest refraction (by inserting an ophthalmic lens of + 0.25 D in the trial frame) to adjust far vision measurements to infinity. Near VA was tested using the same optotype, placed also at 3.5 m, and adding a negative lens to simulate the near distance. The power of the negative lens was determined according to the conditions established in each experiment. All measurements were taken monocularly with the natural pupil. IOLMaster (Carl Zeiss Meditec, Jena, Germany) was used for optical biometry measurements, postoperative pupil size was included.

**Table 1:** Sample data of clinical experiments

Implanted IOL group	Subjects (Eyes)	Age (years) mean ± SD (min, max)	IOL power (D) mean ± SD (min, max)	Pupil (mm) mean ± SD (min, max)
*Clinical experiment 1*				
AT LISA tri group 1	20 (20)	65.05 ± 5.28 (54, 74)	21.17 ± 2.92 (14, 26)	3.43 ± 0.34 (2.91, 4.12)
FineVision	20 (20)	63.70 ± 5.23 (54, 72)	20.72 ± 3.06 (19.0, 23.5)	3.74 ± 0.60 (3.00, 5.04)
*Clinical experiment 2*				
AT LISA tri group 2	10 (20)	64.70 ± 6.72 (49, 73)	21.13 ± 3.20 (14, 27)	3.51 ± 0.39 (2.9, 4.2)

FineVision subjects were recruited from Presbit (Sabadell) (12) and Creu Groga (Calella) (8). AT LISA tri group 1 subjects were recruited from Presbit (Sabadell) (10) and Creu Groga (Calella) (3), Eurolaser (Mataró) (1), and Hospital de Mataró (Mataró) (6). AT LISA tri group 2 subjects were recruited from Hospital de Mataró (Mataró) (10). All centers are in the province of Barcelona, Spain

*IOL* = intraocular lens; *SD* = standard deviation

The refractive correction obtained under W light was used throughout the assessment. The background luminance of the optotypes was 25.3 ± 0.1 cd/m^2^, constantly controlled with a Mavolux 5032C photometer. The room was kept in mesopic conditions to avoid any interference with the measurements. A set of high contrast optotype charts were designed for the purpose of our investigation in accordance with the recommendations of the Universal Ophthalmological Council of 1984 [30] and the guidelines of the ISO 8596:2018 [31]. They are further described elsewhere [32] and were printed with high-resolution quality. The stimulus size of the successive lines followed decimal progression in 0.1 steps. Unlike the logarithmic progression, this design—ISO 8596:2018 compliant—permitted smaller increments in the stimulus size, allowing us to detect finer VA variations [32] in the vicinity of 0.0 logMAR. The order of the background colour presentation was randomized, and subjects were only prompted once for each VA measure. The last visual level where the subject correctly called 3 stimuli out of the 5 presented in the same line was taken as the criterion for determining the VA grade (ISO 8596:2018) [31]. Decimal VA values were converted to the logMAR equivalent values for data processing, statistical analysis, and presentation.

Although the observational data were oriented to obtain evidence of physical facts and the enrolment of a few subjects would have likely sufficed, we decided to recruit more subjects to conform groups of conventional size in this type of studies. Statistical analysis was performed using SPSS software version 13.0 (SPSS Inc., Chicago, IL, USA). Descriptive statistics—mean ± standard deviation (SD)—characterized the sample. The Kolmogorov-Smirnov test did not confirm the normal distribution of data. The Wilcoxon test was applied to paired data to assess the VA differences. For independent data, the Mann-Whitney U test was used. A difference was considered statistically significant for a *P* value less than 0.05.

## Results

### Optical experiment

**Fig. 2:**
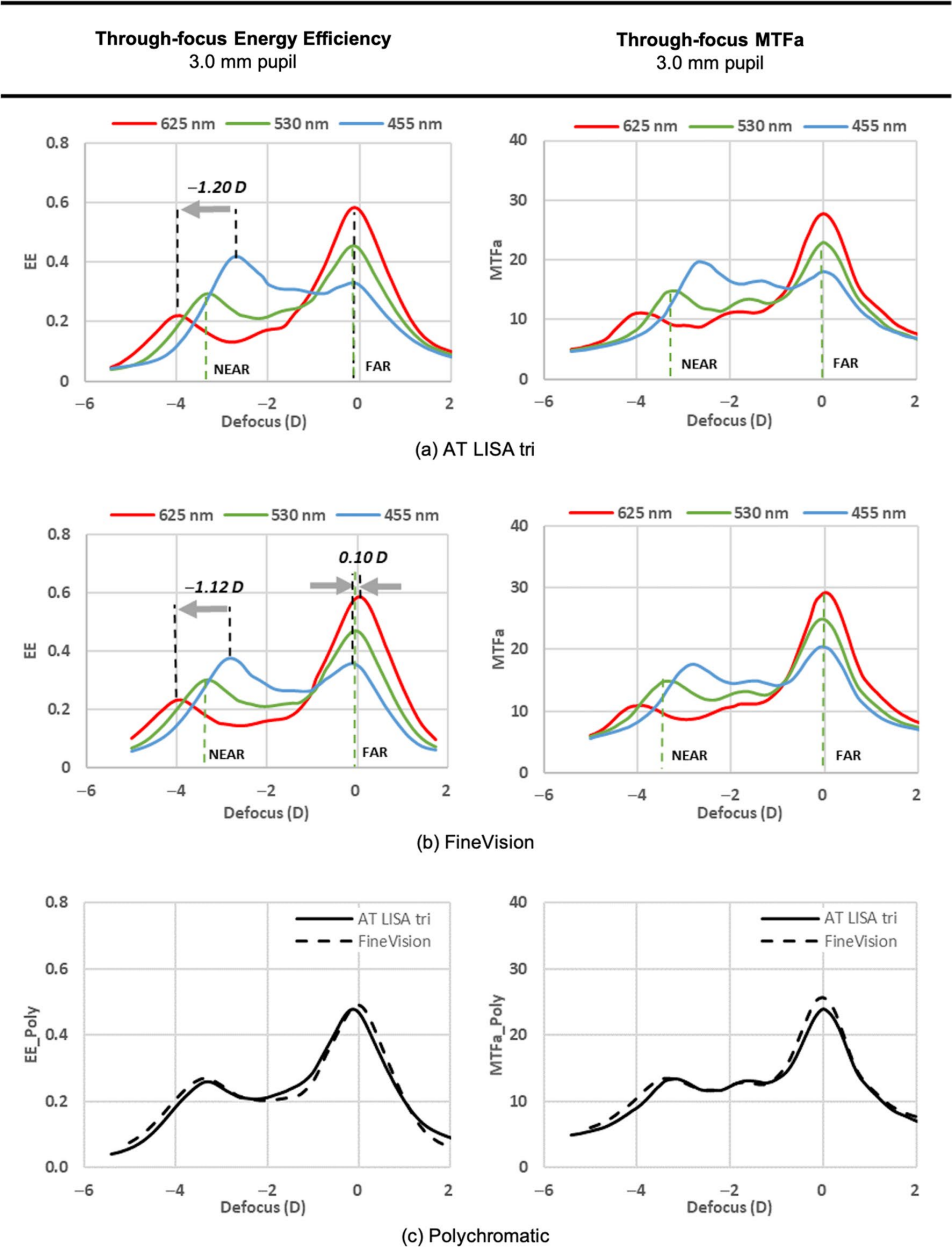
TF-EE and TF-MTFa measurements obtained in the laboratory experiment for the trifocal diffractive AT LISA tri (**a**) and FineVision (**b**) IOLs under R, G, B lights and 3.0 mm pupil; **c** Polychromatic TF-EE and TF-MTFa curves of both IOLs. TF-EE, through-focus energy efficiency; TF-MTFa, through-focus area under the modulation transfer function; R, red; G, green; B, blue; IOL, intraocular lens

Figure 2 shows the TF-EE and TF-MTFa results obtained with the 3.0 mm pupil and the R, G, B lights, whereas Additional file 1: Fig. S5 shows the same for the 4.5 mm pupil. The polychromatic TF-EE_poly_ and TF-MTFa_poly_ plots (Fig. 2c and Additional file 1: Fig. S5c), were computed from the experimental R, G, B TF-EE and TF-MTFa curves weighted by the coefficients that would generate (6500 K) W light.

Figure 2 shows that both IOLs have two clear foci, for far and near vision. Between them, EE and MTFa metrics decrease smoothly, yet with a certain trend of recovery for intermediate distances. The curves corresponding to the simulated polychromatic W light are very close to those measured under G (530 nm) illumination.

LCA was measured for each IOL (Fig. 2 and Table 2). Since the on-bench eye model had an achromatic doublet for the artificial cornea, the LCA values can be considered as due to the IOL. LCA was very small and hardly measurable in the far focus of the two IOLs, but it exceeded 1.0 D (negative) in the near focus.

**Table 2:** Longitudinal chromatic aberration for the far and near IOL foci, obtained from the through-focus energy efficiency values of Fig. 2 (3.0 mm pupil)

Intraocular lens	Longitudinal chromatic aberration	
	Far focus (D)	Near focus (D)
AT LISA tri	0.00 ± 0.10	− 1.20 ± 0.10
FineVision	0.10 ± 0.10	− 1.12 ± 0.10

The spectral range covers from the blue to the red light emitting diode lights (455 to 625 nm)

**Fig. 3:**
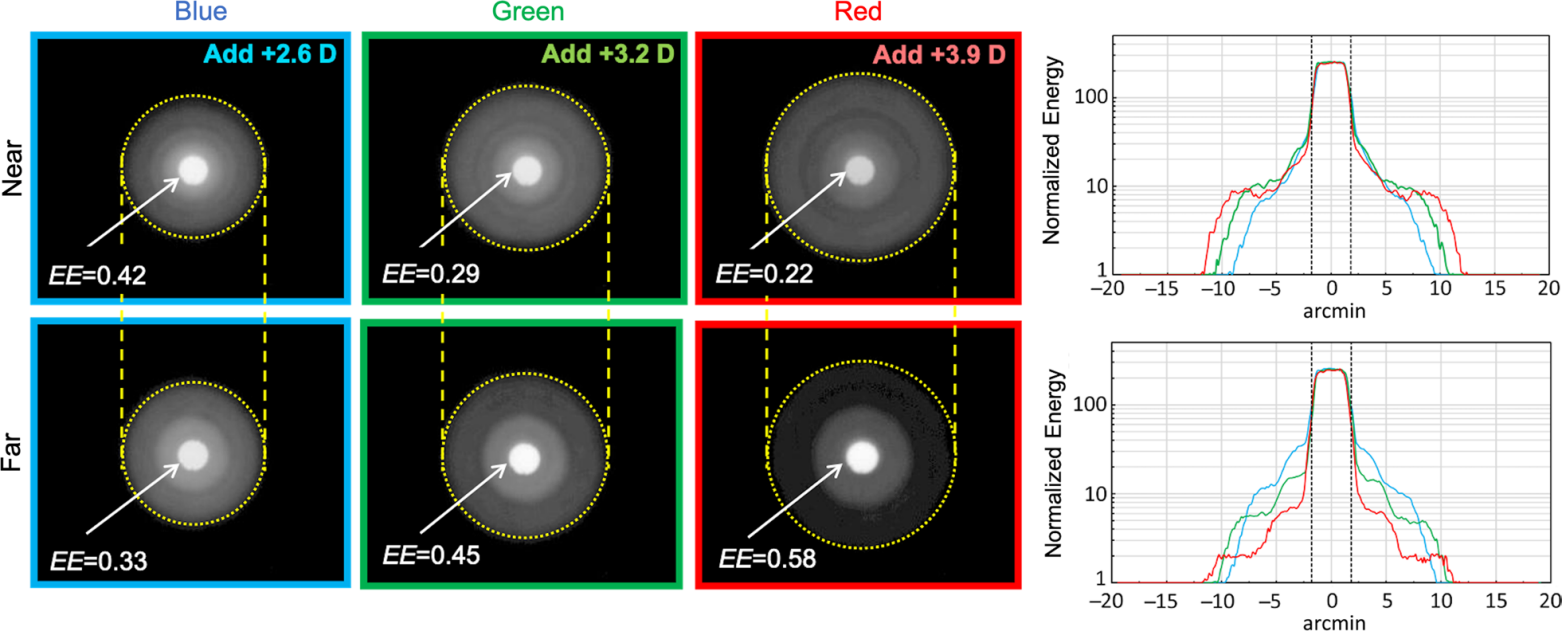
Red (R), green (G), blue (B) images of a pinhole test at near and far foci for AT LISA tri, 3.0 mm pupil. Energy efficiency (EE) values are provided. In the near focus (top row), the add power (D) is given for R, G, B lights. Front halo images and their halo profiles (right most panel) are presented in logarithmic scale of intensity for the sake of visibility

Quite importantly for our study, the distribution of EE between the lens foci changes remarkably with wavelength (Fig. 2, left column), as it can be expected from the optical path differences introduced by the diffractive step height in wavelengths other than the design [8, 33]. Thus, the R and B curves of the TF-EE differ clearly in opposite directions from the G curve: while the R light greatly benefits the far focus to the detriment of the near, the B light benefits the near focus at the expense of the far. The wavelength dependence of both the optical power and EE influences the contrast and size of the simultaneous images formed at the focal planes, with only one image being focused at any one time with the rest being out-of-focus. This effect is illustrated in Fig. 3 for the AT LISA tri. In the far focus (bottom row), the R image shows the best contrast (highest intensity in the image core and lowest intensity in the surrounding halo), followed by the G image and the B image, the latter showing the worst contrast. In the near focus (top row), however, the energy distribution is the opposite: the R image shows the worst contrast, closely followed by the G image, and the B image shows the best. Moreover, the size of the haloes, determined basically by the out-of-focus images [34], depends on the addition power existing between the far and near foci, which, in turn, depends on the wavelength. Thus, the largest halo corresponds to the highest add power (3.9 D, R light; Fig. 3), whereas the smallest halo corresponds to the lowest add power (2.6 D, B light).

**Fig. 4:**
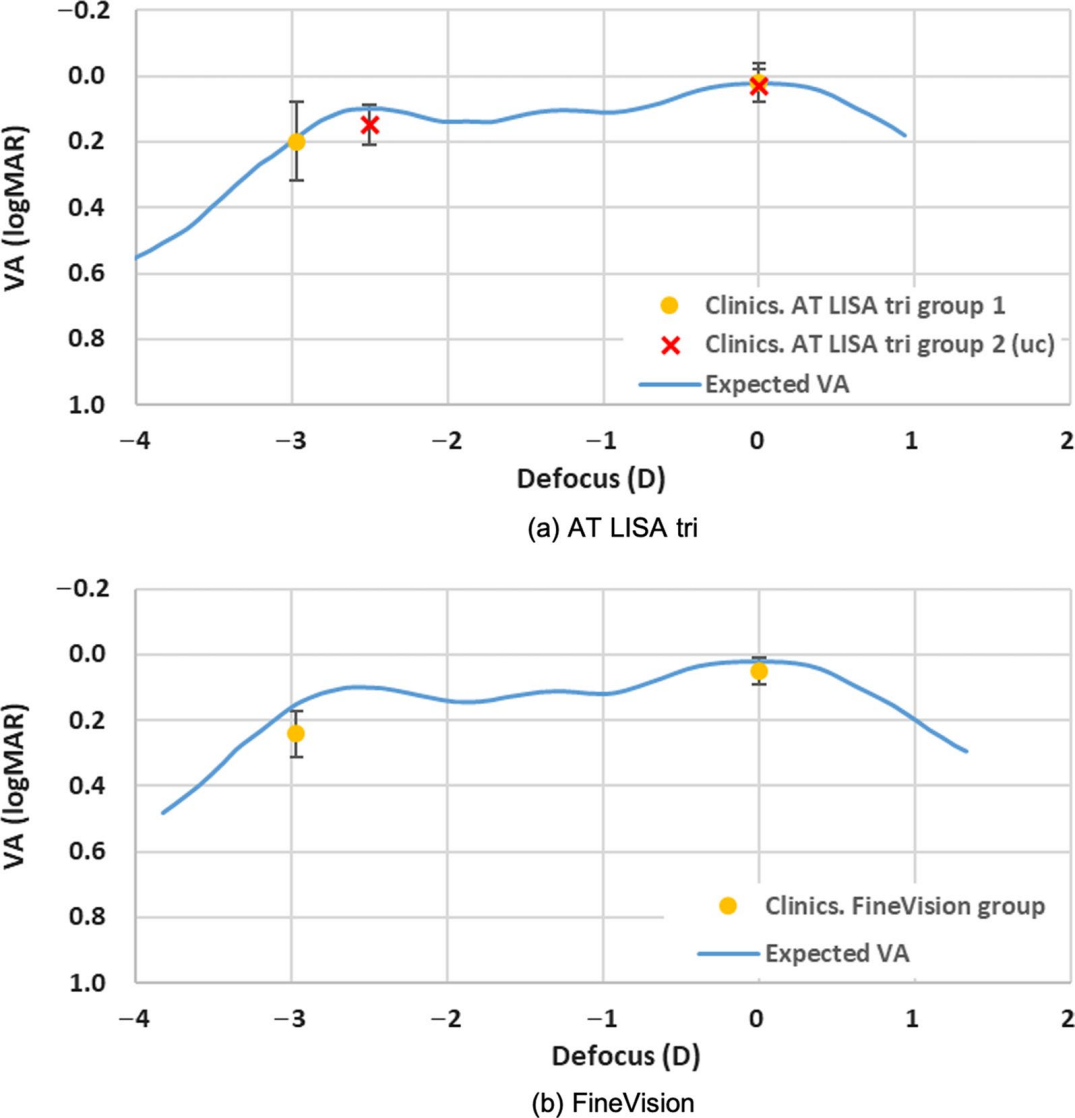
Expected visual acuity (VA, logMAR) defocus curves (blue lines) of patients implanted with AT LISA tri (**a**) and FineVision (**b**) IOLs under (6500 K) white LED illumination. Actual clinical assessments (mean ± SD) of pseudophakic patients with best distance correction are represented by dots and error bars. In experiment 1, patients enrolled in the AT LISA tri group 1 and FineVision group were assessed in far and (− 3 D) near vision (yellow dots). In experiment 2, patients of AT LISA tri group 2 were further assessed in far and best near vision (red crosses). IOL, intraocular lens; LED, light emitting diode; uc, uncompensated longitudinal chromatic aberration

We calculated the expected VA (logMAR) of the pseudophakic patients under W illumination from the TF-MTFa_poly_ (calculated, in turn, from the R, G, and B TF-MTFa measurements, Fig. 2) taken with a 3.0 mm pupil [3]. Figure 4 shows the expected defocus curves (blue line) for the AT LISA tri and FineVision IOLs. They predict a very good postoperative VA, close to 0.0 logMAR in far vision, which decreases smoothly in intermediate vision with some improvement in near. Defocus is represented at the spectacle plane [35] in Fig. 4. Taken together, a sustained good visual quality (equal or better than 0.2 logMAR) can be expected for the average patient in a depth-of-focus range that goes from infinity to roughly 30 cm from the subject (− 3.0 D defocus). This prediction is intended for comparison with the actual clinical VA outcomes as explained in the clinical experiments.

### Clinical experiment 1

**Fig. 5:**
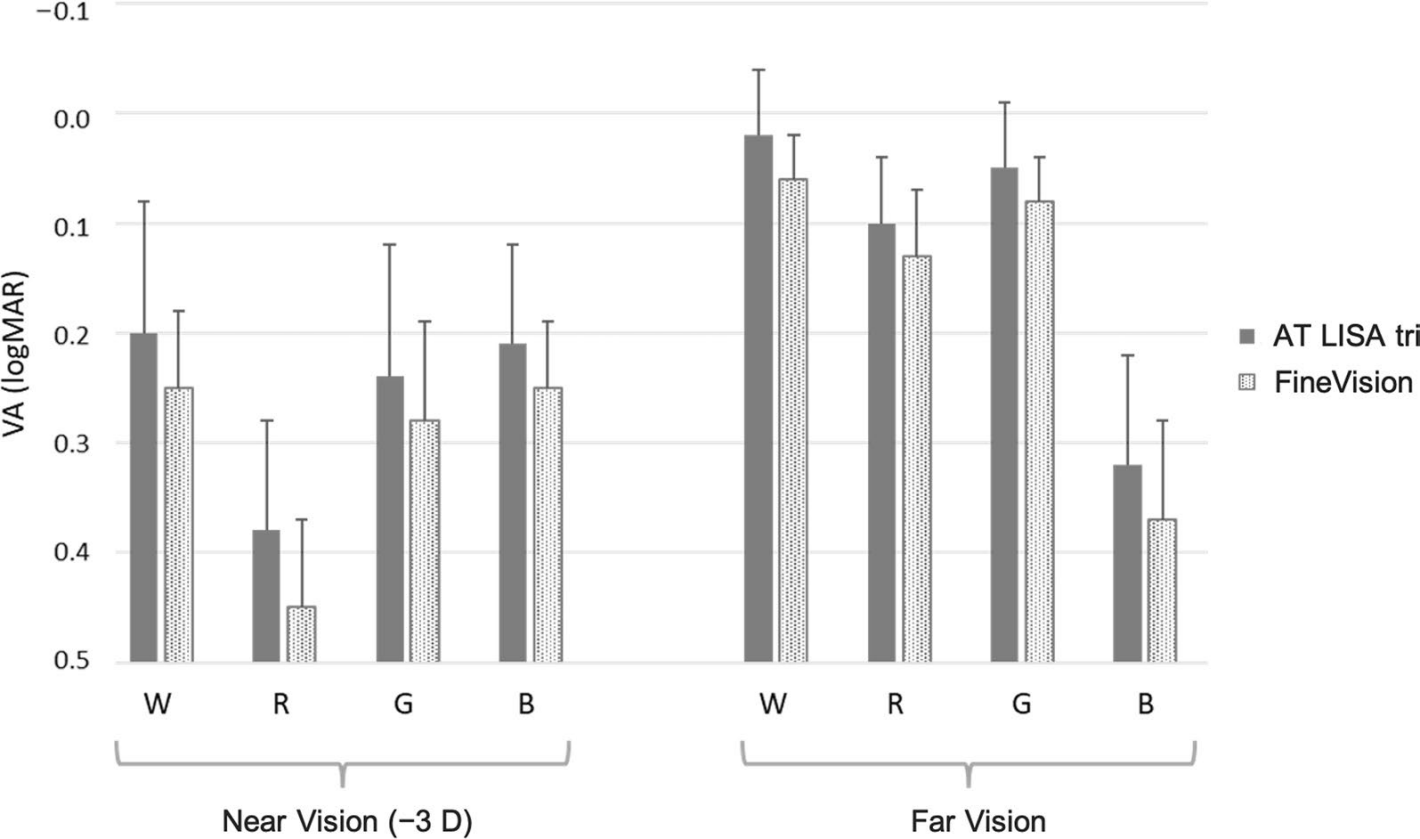
Mean visual acuity (VA, logMAR) values and standard deviation segments reached by two groups of pseudophakic patients at far and near vision under W, R, G, and B illumination (clinical experiment 1). W, white; R, red; G, green; B, blue

VA of pseudophakic subjects was tested at two fixed distances—far (0.0 D) and near (− 3.0 D), under successive W, R, G, and B illumination (Fig. 1a, b). Figure 5 shows the average VA outcomes obtained for the FineVision group and the AT LISA tri group 1. The VA results include the joint effects of the EE wavelength dependence of the diffractive IOL and the LCA of the pseudophakic eye. The mean VA outcomes are consistently similar for both trifocal IOLs under all four illuminations although slightly better for subjects with AT LISA tri. Despite the presence of LCA, the VA reached under W light was equal or better than any other colour light in both the far and near vision conditions. The (mean ± SD) values and their statistical significance are given in Tables 3 and 4, respectively. The mean VA values with W illumination are represented with yellow dots in Fig. 4. It is worth remarking the excellent agreement with the predicted values for the AT LISA tri group. The prediction for the FineVision group slightly overestimated the clinical results. In general, the mean VA values are better in far than in near vision for all the illumination conditions, except for the B light (Fig. 5 and Additional file 1: Fig. S6). Moreover, at near distance, the VA with B illumination is as good as the VA achieved with W light, with non-statistically significant difference for both the AT LISA tri (*P* = 0.57) and FineVision (*P* > 0.99) groups (Table 4). With R illumination, the visual quality worsens severely in near vision, more than with W and G lights (Fig. 5, Table 3, and Additional file 1: Fig. S6). Note that the good VA achieved at far distance under R light (0.10 ± 0.06 logMAR for AT LISA tri, 0.13 ± 0.06 logMAR for FineVision) drops off dramatically to the worst VA at near (0.38 ± 0.10 logMAR for AT LISA tri, 0.44 ± 0.08 logMAR for FineVision), even worse than the poor VA outcomes obtained at far distance under B light (0.32 ± 0.10 logMAR for AT LISA tri, 0.37 ± 0.09 logMAR for FineVision).

**Table 3:** Visual acuity (logMAR) values (mean ± SD) obtained in clinical experiment 1 for far (0.0 D) and near (− 3.0 D) vision

Group	Pupil (mm)	Visual acuity (logMAR)							
		Near				Far			
		W	R	G	B	W	R	G	B
AT Lisa tri group 1	3.43 ± 0.34	0.20 ± 0.12	0.38 ± 0.10	0.24 ± 0.12	0.21 ± 0.09	0.02 ± 0.06	0.10 ± 0.06	0.05 ± 0.06	0.32 ± 0.10
FineVision	3.74 ± 0.60	0.24 ± 0.07	0.44 ± 0.08	0.28 ± 0.09	0.24 ± 0.06	0.05 ± 0.04	0.13 ± 0.06	0.08 ± 0.04	0.37 ± 0.09

*W* = white; *R* = red; *G* = green; *B* = blue

**Table 4:** *P* value for the mean pairwise comparison (Wilcoxon test) of the visual acuity obtained under W, R, G, B lights at far (0.0 D) and near (− 3.0 D) vision for the (AT LISA tri / FineVision) IOLs

	Near vision				Far vision			
	W	R	G	B	W	R	G	B
W	–	< 0.01	< 0.01	(0.57* / > 0.99*)	–	< 0.01	0.02	< 0.01
R	–	–	< 0.01	< 0.01	–	–	< 0.01	< 0.01
G	–	–	–	(0.07*/ 0.03)	–	–	–	< 0.01
B	–	–	–	–	–	–	–	–

*W* = white; *R* = red; *G* = green; *B* = blue

Values common to both lenses appear only once. Asterisk indicates a difference with non-statistical significance (*P* > 0.05)

### Clinical experiment 2

This experiment aims to bring to light the effects of two separate factors: one, the EE wavelength dependence of the IOL foci (determined by the operative diffractive orders) and, the other, the LCA of the pseudophakic eye. We want also to evaluate their relative influence on either far and near vision, as well as to emphasize the possible differences in comparison with the natural phakic human vision.

The experiment consisted of four stages (Fig. 1c) in the VA assessment: two concerned far vision with uncompensated (uc) LCA (stage 1) and compensated (c) LCA (stage 2), and the other two concerned near vision with uncompensated (uc) LCA (stage 3) and compensated (c) LCA (stage 4). For the LCA compensation of every subject in each illumination condition, we used additional trial ophthalmic lenses, with the (± 0.25 D) uncertainty assumed in ordinary clinical examinations.

Note that stage 1 coincided with the first part of the experiment 1, but it was applied to a new group of subjects (namely, AT Lisa tri group 2). In stage 3 (near vision with uncompensated LCA), we determined the refractive addition for the best near distance corrected VA under W light and this refractive addition was kept unchanged with the other illuminations. LCA was individually compensated under R, G, B lights at far vision in stage 2 and at near vision in stage 4.

**Fig. 6:**
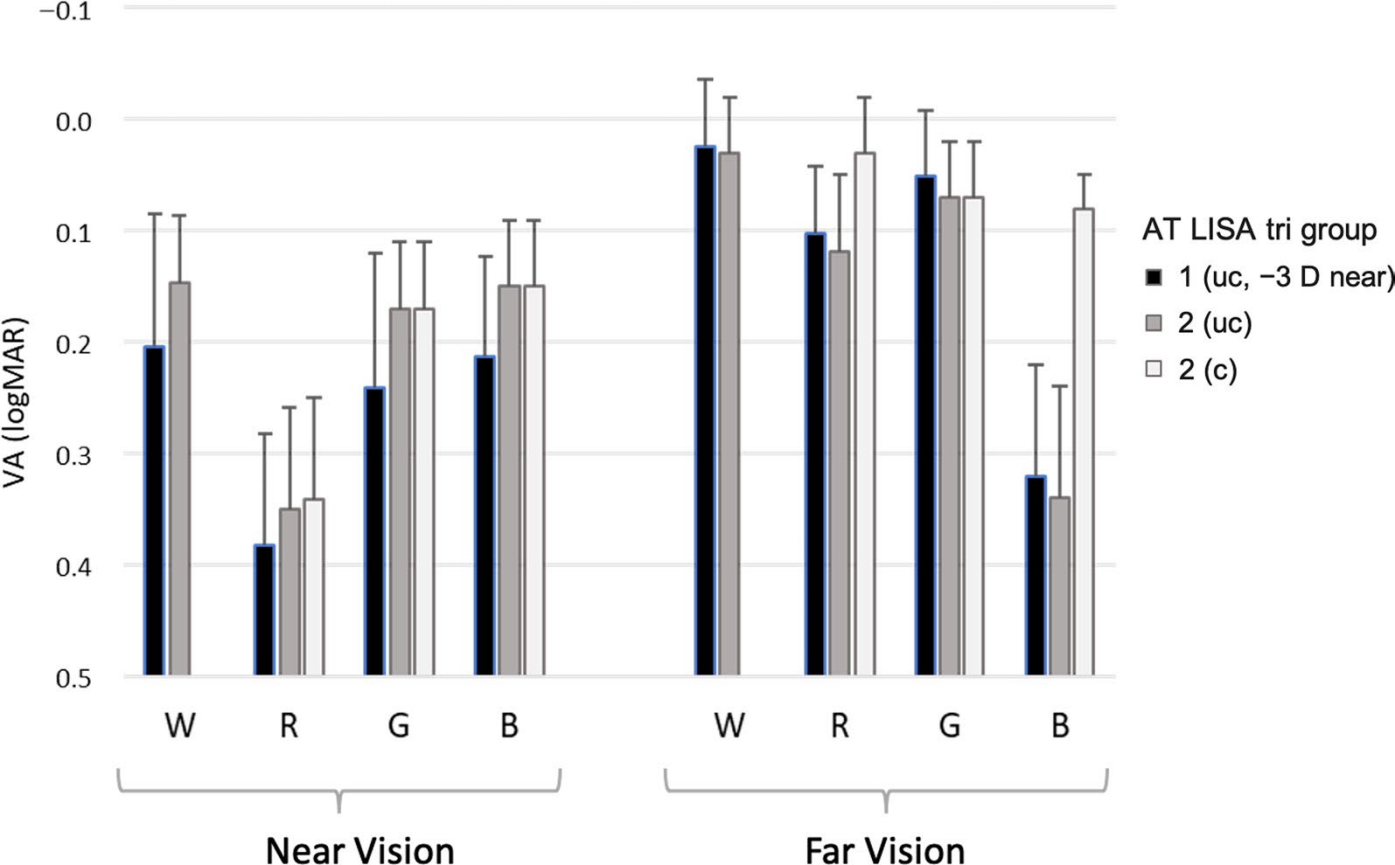
Mean visual acuity (VA, logMAR) values and standard deviation segments reached by the AT LISA tri groups at far and near vision under W, R, G, and B lights in the clinical experiments 1 and 2. W, white; R, red; G, green; B, blue; uc, uncompensated longitudinal chromatic aberration (LCA); c, compensated LCA

For the sake of comparison, we present the results (mean ± SD) of the VA outcomes for the AT LISA tri groups 1 and 2 in Fig. 6 and Table 5. The VA values of the AT LISA tri group 1 were obtained with uncompensated (uc) LCA and constant addition of − 3.0 D at near vision.

**Table 5:** Visual acuity (logMAR) values (mean ± SD) obtained in clinical experiment 2 for far and near vision

AT Lisa tri subjects	Pupil (mm)	Visual acuity (logMAR)							
		Near				Far			
		W	R	G	B	W	R	G	B
Group 1 (uc)	3.43 ± 0.34	0.20 ± 0.12	0.38 ± 0.10	0.24 ± 0.12	0.21 ± 0.09	0.02 ± 0.06	0.10 ± 0.06	0.05 ± 0.06	0.32 ± 0.10
Group 2 (uc)	3.51 ± 0.39	0.15 ± 0.06	0.35 ± 0.09	0.17 ± 0.06	0.15 ± 0.06	0.03 ± 0.05	0.12 ± 0.07	0.07 ± 0.05	0.34 ± 0.10
Group 2 (c)	3.51 ± 0.39	–	0.34 ± 0.09	0.17 ± 0.06	0.15 ± 0.06	–	0.03 ± 0.05	0.07 ± 0.05	0.08 ± 0.03
		*Refractive compensation (D) for Group 2*							
Group 2 (uc)		− 2.53 ± 0.11	− 2.53 ± 0.11	− 2.53 ± 0.11	− 2.53 ± 0.11	0.00 ± 0.00	0.00 ± 0.00	0.00 ± 0.00	0.00 ± 0.00
Group 2 (c)		–	− 2.56 ± 0.09	− 2.53 ± 0.11	− 2.53 ± 0.11	–	0.26 ± 0.15	0.00 ± 0.00	− 0.75 ± 0.11

(c) and (uc) stand for compensated and uncompensated LCA condition, respectively. The results of the AT LISA group 1, obtained in clinical experiment 1 (with near vision fixed at − 3.0 D), are copied in the first row (group 1(uc)) and depicted in Fig. 6 for comparison. In the last two rows, refractive compensation (D) (mean ± SD) indicates the variation with respect to the best far vision (0.0 D) under W light

*W* = white; *R* = red; *G* = green; *B* = blue

In far vision, the AT LISA group 2 (uc) showed, on average, slight hypermetropia under R light (0.26 ± 0.15 D) and moderate myopia under B light (− 0.75 ± 0.11 D). This chromatic difference of refraction was due to the LCA of the pseudophakic eye as a whole and was still similar to the natural LCA of a phakic eye [5]. After proper correction with ophthalmic lenses, they improved their far VA under these colour lights [Fig. 6 and Table 5, group 2 (c)]. Therefore, under R light and with correction of 0.26 ± 0.15 D, they improved from 0.12 ± 0.07 logMAR to 0.03 ± 0.05 logMAR, matching VA under W light; and, more importantly, under B light and with correction of − 0.75 ± 0.11 D, they improved from 0.34 ± 0.10 to 0.08 ± 0.03 logMAR.

The AT LISA group 2 required − 2.53 ± 0.11 D at the spectacle plane (AT LISA has + 3.33 D design add power at the IOL plane) for best near vision under W light. The mean VA values under W light of group 2 (uc) in far and near vision are in excellent agreement with the expected values predicted from the MTF measurements in the optical experiment, as it can be seen in Fig. 4a.

Group 2 (c) with LCA compensated near vision showed worse VA outcomes in general than uncompensated far vision, except for—and this fact is quite remarkable (explained in the discussion section)—under B light. Near VA under B and W lights matched to 0.15 ± 0.06 logMAR (no matter the compensation). In fact, there was no need for further LCA correction in near vision. Only 3 (15%) individuals out of 20 eyes tested experienced a tiny improvement in their VA under R light after a slight ophthalmic correction of − 0.25D, which, in turn, falls within the uncertainty value. The mean VA at near under R light worsened drastically to 0.35 ± 0.09 logMAR and kept almost unchanged (0.34 ± 0.09 logMAR) despite the allowance for compensation. Table 6 contains the *P* value for the mean pairwise comparisons. It is worth noting that the repeatability of the VA results obtained for the AT LISA groups 1 and 2 (uc) in far vision and similar illumination: the differences between the far VA pairs did not exceed 0.02 logMAR (Table 5). However, the discrepancy between the AT LISA groups 1 and 2 (uc) was larger in near vision, with differences up to 0.07 logMAR for G light (Table 5), very likely because near vision was set at − 3.0 D for the subjects of group 1 whereas, for group 2 (uc), it was determined from the best individual near distance under W light (− 2.53 D on average). Overall, no significant statistical differences were eventually obtained between groups 1 and 2 (uc), neither in far nor in near vision, for all the four illumination conditions (Table 6).

**Table 6:** *P* value for the mean pairwise comparison for the visual acuity obtained under W, R, G, B lights at far and near vision for the AT LISA groups

AT LISA tri subjects	Near vision				Far vision			
	W	R	G	B	W	R	G	B
*Groups 1 and 2 (uc)	0.10	0.32	0.12	0.06	> 0.99	0.46	0.58	0.46
^†^Groups 2 (uc) and 2 (c)	> 0.99	0.10	> 0.99	> 0.99	> 0.99	** < 0.01**	> 0.99	** < 0.01**

*W* = white; *R* = red; *G* = green; *B* = blue

*Mann-Whitney U test; ^†^Wilcoxon test

Statistically significant differences (*P* < 0.05) are in boldface

The comparison between groups 2 (uc) and (c) is more revealing and powerful. LCA compensation turned out to be significant (*P* < 0.05) in far vision under R light and, particularly important, under B light, for which VA improved very remarkably from 0.34 ± 0.10 to 0.08 ± 0.03 logMAR. No significant improvement with LCA compensation can be reported, however, in near vision under any illuminant (*P* > 0.05).

We acknowledge as a possible limitation in the comparison of groups 2 (uc) and (c) that data from both eyes of each subject were used in the clinical experiment 2. This fact artificially reduces the variance between data points and may increase the risk of Type I error (false positive). This would be particularly problematic if the *P* values were near the 0.05 cut-off which is not the case (see Table 6, second row).

## Discussion

We have conducted a series of experiments with two diffractive IOLs (AT LISA tri and FineVision) using two approaches, the in vitro optical-bench and the in vivo clinical methodologies, oriented to answer the questions: How does a multifocal diffractive IOL alter the spatio-chromatic vision? Is the LCA the single reason for the variations in the VA when the object distance and colour light change? Are these alterations consistent with the high tolerance of natural human vision to LCA?

Overall, our in vitro results are in good agreement with former reports [27, 36, 37]. For the FineVision IOL, with the 3.0 mm pupil and under G light, the EE percentages driven at the {far, intermediate, near} foci were {47, 22, 30} out of the total diffracted energy. These values are consistent with the trifocal diffractive design of the lens and close to the theoretical {49, 17, 34} and experimental {51,18, 31} percentages derived from TF-MTF at 50 c/mm reported by Gatinel et al. [27] for a 20.5 D lens with 3.0 mm pupil under 546 nm light. The AT LISA tri also shows trifocal performance and EE distribution ({45, 24, 29} under 530 nm) similar to the FineVision’s.

The MTF and EE measurements obtained in an optical-bench model eye with an achromatic cornea lens allow us to objectively prove how the dominance varies with object distance and colour: red dominance in far, blue dominance in near (Fig. 2). They further allow us to confirm that, in far vision (0th diffraction order), there is a tiny positive contribution—yet within our experimental uncertainty—of the IOLs (Abbe number 58) to ocular LCA due to the material dispersion, whereas in near vision (1^st^ diffraction order, basically), such a contribution is over 1.0 D in magnitude and negative, meaning opposite sign to the LCA of the natural cornea and ocular humours (Table 2 and Additional file 1: Table S2). This fact predicts that, after IOL implantation, the LCA of the ocular media will likely be compensated in near vision but will remain in far vision. However, since in far vision, the amount of LCA would still be very close to the natural one [5], it should be tolerated by the visual system, and the issue is not expected to be clinically relevant.

The differences in LCA for far and near foci of the IOLs are a consequence of the chromatic difference of addition power [8]. It also affects the halo sizes depicted in Fig. 3: the B light leads to lower add power and, hence, to show smaller halo sizes than R light. Although, in general, only the presence of haloes in far vision draws clinical interest, they are also present in near vision (with the same size) and all of them contribute to contrast reduction. From the in vitro R, G, B TF-MTFa measurements, we calculated the expected VA (defocus curve) of pseudophakic patients under white (6500 K W LED) illumination (Fig. 4).

In addition to LCA, the chromatic difference of EE for the 0th and 1st diffraction orders influences the far and near vision in different ways [8] so their effects deserve detailed analysis. It should be noted that, under R light, the two IOLs split much more energy to the far focus than to the near (Fig. 2). Such unbalanced distribution of energy between the co-axial far and near foci leads to a R image with very good contrast in the far focus but low contrast in the near focus (Fig. 3). However, under B light, a rather balanced distribution of energy occurs between the far and near focus (FineVision IOL) or even with a far focus less intense than the near (AT LISA tri) (Fig. 2). In this case, the B image has similar contrast in both foci, and even better in the near than in the far focus (AT LISA tri, Fig. 3). These facts lead us to predict uncommon asymmetrical variations in the mean VA under R and B lights when the object distance changes.

The results of the two clinical experiments conducted in this study allow us to confirm:

- Under W light, the clinical mean VA values are in excellent agreement with the predicted values of the defocus curves, calculated from the TF-MTFa measurements of the two IOLs (AT LISA tri and FineVision) (Fig. 4), for three vision distances: far (0.0 D), near (set to − 3.0 D), and best near (− 2.53 ± 0.11 D, AT LISA tri in Table 5). The VA values under W light in far vision (about 0.0 logMAR) are consistent with the results reported by others [38–40].
- Under G light, the clinical mean VA values are very close—but slightly worse—than those obtained under W light, for all the IOLs and the far/near distance conditions (Tables 3 and 5). Such proximity of the mean VA values under W and G lights lies in the fact that the G light approaches the design wavelength (546 nm [19]), which is in the vicinity of the maximum photopic efficiency of humans. Although LCA was nearly eliminated by using G illumination, the visual benefit of solely suppressing LCA is unnoticeable in phakic human vision because monochromatic aberrations cause a larger reduction in visual quality [41–43]. This fact was formerly reported for monofocal pseudophakic subjects as well [44].
- Evidence of spatio-chromatic changes in pseudophakic vision with diffractive IOLs are provided in this study. They are a direct consequence of the diffractive nature of the add power profile of the IOLs. We evaluated those changes through the mean VA outcomes when changing the spectral wavelength band of the light and the vision distance. Thus, the negative LCA in the near focus of the IOLs (Fig. 2 and Table 2, Additional file 1: Fig. S5, and Table S2) has significative compensating effects on the positive LCA of the ocular media [*P* > 0.05 in Table 6 for the AT LISA groups 2 (uc) and 2(c) in near vision with all the W, R, G, B lights]. Such LCA compensation in near vision explains that the subjects, with the ophthalmic correction corresponding to their best near vision under W light, did not require any further correction when illuminated with either B or R lights, except for a minimal additional correction of − 0.25 D in 3 eyes (15%) out of 20 (Fig. 6 and Table 5). In contrast, the same subjects experienced the effects of the LCA aberration on far vision because, in this focus, the IOL did not compensate for the remaining LCA of the rest of the ocular media, but instead, contributed with some tiny positive LCA due to the material dispersion. For this reason, subjects with best distance corrected vision under W light significantly improved their VA in far vision under B and R illuminations after further refractive compensation for LCA (*P* < 0.01, Table 6). Hence, under B light and with an ophthalmic compensation of − 0.75 ± 0.11 D, they greatly improved their VA at far, from 0.34 ± 0.10 to 0.08 ± 0.03 logMAR. Under R light and with an ophthalmic compensation of 0.26 ± 0.15 D, subjects experienced a more modest improvement of VA at far, from 0.12 ± 0.07 to 0.03 ± 0.05 logMAR.
- The visual quality under R and B lights shows asymmetry between near and far vision (Fig. 6, Tables 5 and 6). This asymmetry cannot be explained solely from the LCA effects, which, in turn, does not have a crucial impact on visual perception under W light. Thus, subjects implanted with the AT LISA tri, under R illumination, have much worse VA in near (0.35 ± 0.09 logMAR) than in far vision (0.12 ± 0.07 logMAR). As we have demonstrated, their VA at near under R light does not significantly improve with LCA compensation (0.34 ± 0.09 logMAR, *P* = 0.10). The opposite happens under B light, which, in comparison with the other W, R, G illuminations, leads to the worst VA outcomes in far vision (0.34 ± 0.10 logMAR) but the best in near vision (0.15 ± 0.06 logMAR). Although after LCA compensation in far vision, subjects significantly improved their VA under B light (0.08 ± 0.03 logMAR), they did not reach the VA under R light (0.03 ± 0.05 logMAR, which matched the VA under W light). In near vision, however, subjects had a VA under B light as good as under W light (0.15 ± 0.06 logMAR) and much better than under R light (0.34 ± 0.09 logMAR). The reason for these uncommon facts in near vision is found in the wavelength dependence of the EE of diffractive components, evidenced in the asymmetric distributions of energy between the far and near foci under R and B illuminations (Fig. 2 and Additional file 1: Fig. S5). Finally, our results are consistent with those obtained by Łabuz et al. [20] with a red filter and give a complete explanation to them.

The last two paragraphs report the most distinct findings of our study.

## Conclusions

We found asymmetric alterations in the spatio-chromatic vision produced by multifocal diffractive IOLs, with significant changes in resolution depending on the object distance and the spectral band of the illumination; these alterations can be detected with the methods, procedures, and materials of the ordinary clinical examination. The alterations depend strongly on the characteristics of the diffractive design, in particular: the diffraction orders used for far vision and presbyopia correction, the pupil in case of apodization or other types of pupil-dependent profile designs, and the spectral band of the illumination.

It is challenging to determine how these changes with the object distance would interfere with the neural response to the wavelength, such as the opponent-colours signals, the receptive field pathways and the neural mechanisms of colour appearance [45]. All these factors play an essential role in tasks related to colour vision such as colour texture matching, edge detection, segmentation and pattern recognition. Further investigation should cover a variety of IOL diffractive designs and determine how these alterations affect visual perception in daily tasks. It will also interest designers of new presbyopia-correcting IOLs.

## Supplementary Information

**Additional file 1: Table S1.** Data of the light sources. Peak wavelength λ, full width at half maximum, correlated colour temperature, and chromatic coordinates in the CIE 1931 colour space chromaticity diagram. **Table S2.** Longitudinal chromatic aberration for the far and near IOL foci, obtained from the through-focus energy efficiency values of Figure S5. **Figure S1**. Schemes of the optical-bench testing setup. **a** Layout with an inset showing multiple foci; **b** Object tests; **c** Opto-mechanic depiction. **Figure S2.** Light sources. **a** to **d** Spectral band emissions of the LED sources: red, green, blue, and white with CCT 6500 K. **e** CIE 1931 colour space chromaticity diagram with the LED points. The straight lines are used to determine the weights of R, G, B mixture to be equivalent to W. **Figure S3**. Metrics for the optical characterization of IOLs in the laboratory experiment. **a** Energy efficiency calculated through the light-in-the-bucket ${E}_{core}/{E}_{total}$ ratio, with ${E}_{total}={E}_{core}+{E}_{backg}$, applied to the image of the pinhole test. **b** Area under the MTF calculated from the line spread function. Scheme of the TF-MTFa measurement. **Figure S4.** Images of the pinhole test formed by the model eye with an IOL immersed: in linear grayscale of intensity, intensity profile, and in logarithmic grayscale. **Figure S5**. TF-EE and TF-MTFa measurements obtained in the laboratory experiment for the trifocal diffractive IOLs. **a** AT LISA tri; **b** FineVision, under R, G, B lights and 4.5 mm pupil; **c** Polychromatic TF-EE and TF-MTFa curves of both IOLs. **Figure S6.** Far to near visual acuity differences under W, R, G, B lights for the subjects implanted with AT LISA tri and FineVision IOLs. Computed from Table 3.

## Data Availability

The datasets used and/or analysed during the current study are available from the corresponding author on reasonable request.

## Abbreviations

B: Blue
c: Compensated
EE: Energy efficiency
G: Green
IOL: Intraocular lens
LCA: Longitudinal chromatic aberration
LED: Light emitting diode
MTF: Modulation transfer function
MTFa: Area under the modulation transfer function curve
poly: Polychromatic
R: Red
SA: Spherical aberration
SD: Standard deviation
TF: Through-focus
uc: Uncompensated
VA: Visual acuity
W: White
