# Supplementary material for: Spatio-chromatic vision with multifocal diffractive intraocular lens
Source: Eye Vis (Lond). 2023 Aug 1;10:32. doi: 10.1186/s40662-023-00350-5 (PMC10391998; doi:10.1186/s40662-023-00350-5)

**Table S1.** Data of the light sources. Peak wavelength λ (nm), full width at half maximum (FWHM), correlated colour temperature (CCT) (K), and chromatic coordinates (x, y) in the CIE 1931 colour space chromaticity diagram.

|  |  |  |  |  |  |
| --- | --- | --- | --- | --- | --- |
| LED | Trademark and model | λ (nm) | FWHM (nm) | CCT (K) | (x,y) |
| W | Thorlabs MCWHL5-LED | - | - | 6500 | (0.3128, 0.3292) |
| R | Thorlabs M625L3-LED | 625 | 18 | - | (0.7017, 0.2981) |
| G | Thorlabs M530L3-LED | 530 | 33 | - | (0.1224, 0.7478) |
| B | Thorlabs M455L3-LED | 455 | 18 | - | (0.1506, 0.0262) |
|  |  |  |  |  |  |

LED = light emitting diode; W = white; R = red; G = green; B = blue

**Table S2.** Longitudinal chromatic aberration for the far and near IOL foci, obtained from the through-focus energy efficiency values of Figure S5 (4.5 mm pupil).

|  | **Longitudinal chromatic aberration** | |
| --- | --- | --- |
| **Intraocular lens** | **Far focus (D)** | **Near focus (D)** |
| AT LISA tri | 0.00 ± 0.10 | −1.30 ± 0.10 |
| FineVision | 0.10 ± 0.10 | −1.02 ± 0.10 |

**Figure S1**. Schemes of the optical-bench testing setup. **a** Layout with an inset showing multiple foci; **b** Object tests; **c** Opto-mechanic depiction.


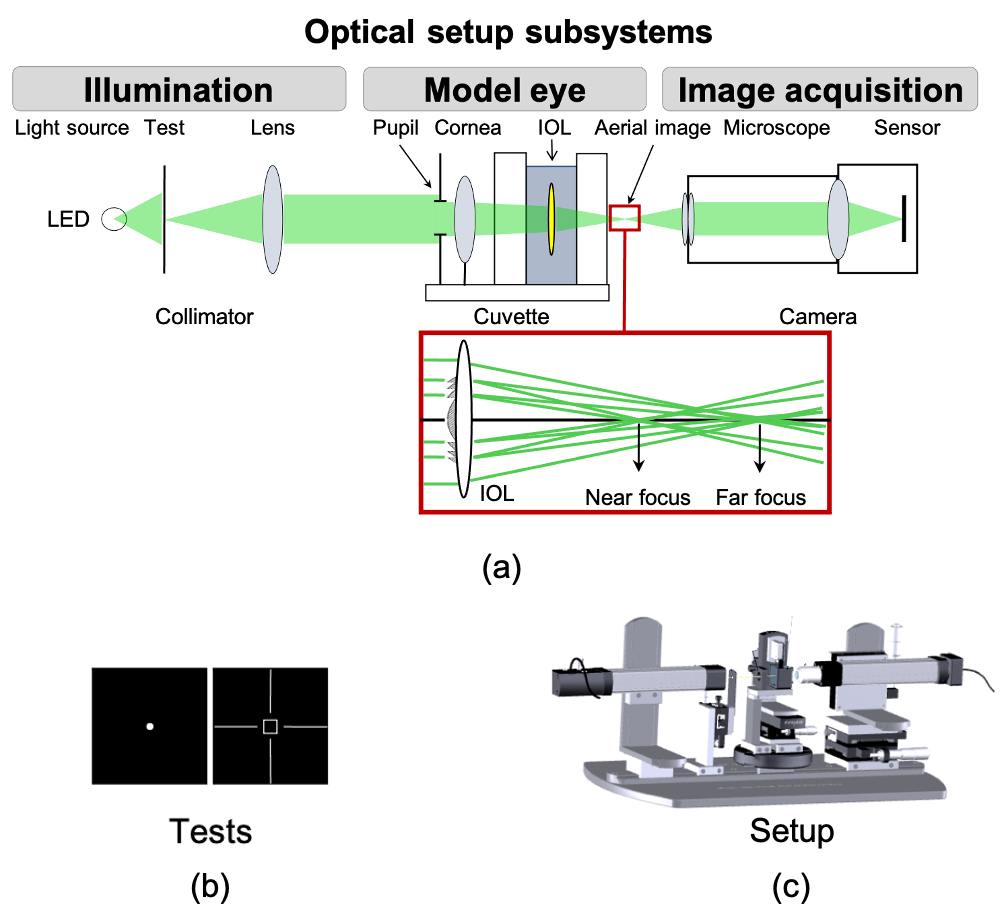


**Figure S2.** Light sources. **a** to **d** Spectral band emissions of the LED sources (Thorlabs GmbH, Munich, Germany): red (R), green (G), blue (B), and white (W) with CCT 6500 K. **e** CIE 1931 colour space chromaticity diagram with the LED points. The straight lines are used to determine the weights of R, G, B mixture to be equivalent to W [25]. LED, light emitting diode


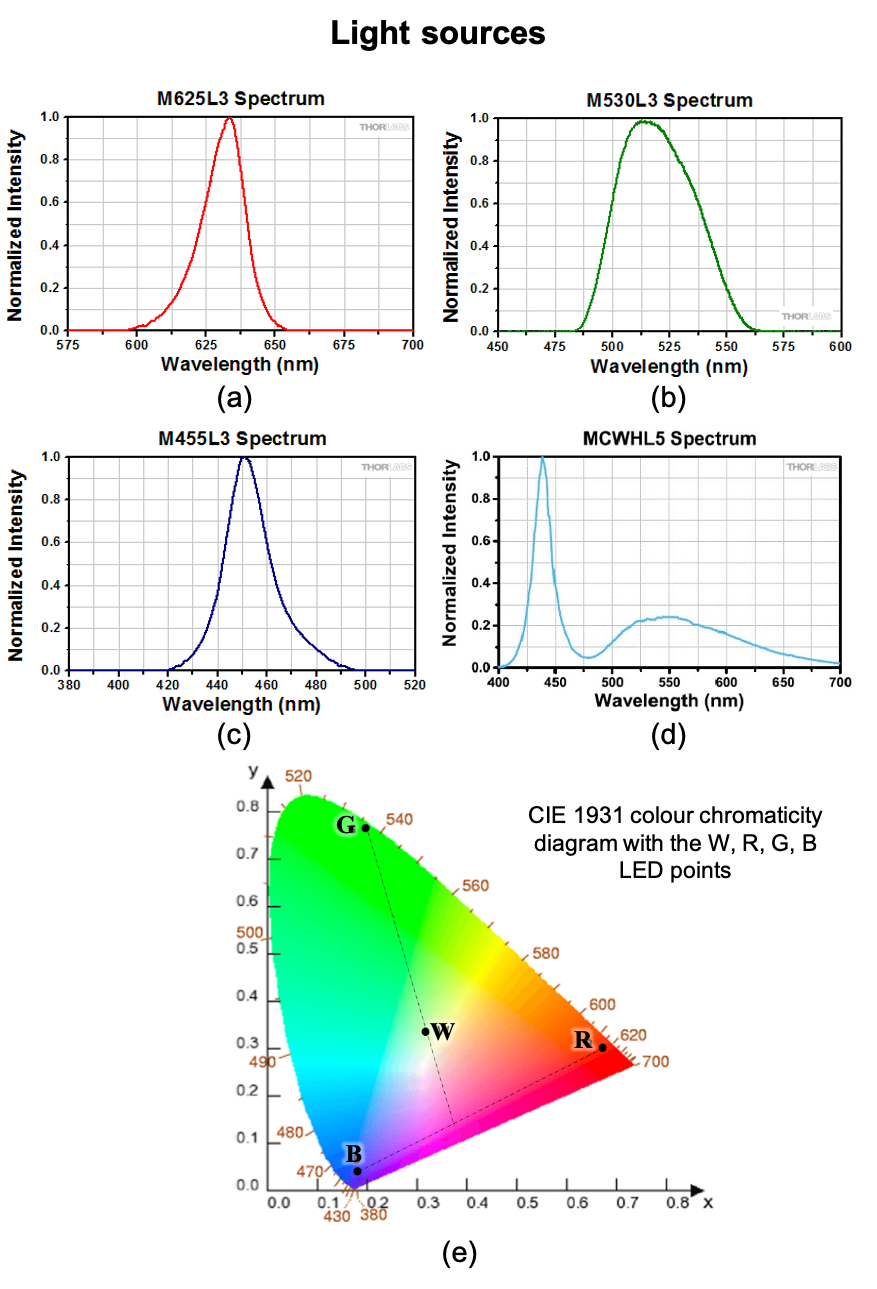


**Figure S3**. Metrics for the optical characterization of IOLs in the laboratory experiment. **a** Energy efficiency (EE) calculated through the light-in-the-bucket ${E_{core}}/{E_{total}}$ ratio, with $E_{total}=E_{core}+E_{backg}$, applied to the image of the pinhole test (backg = background). **b** Area under the MTF (MTFa) calculated from the line spread function (image of the four-slit test). Scheme of the TF-MTFa measurement. TF, through-focus; MTF, modulation transfer function


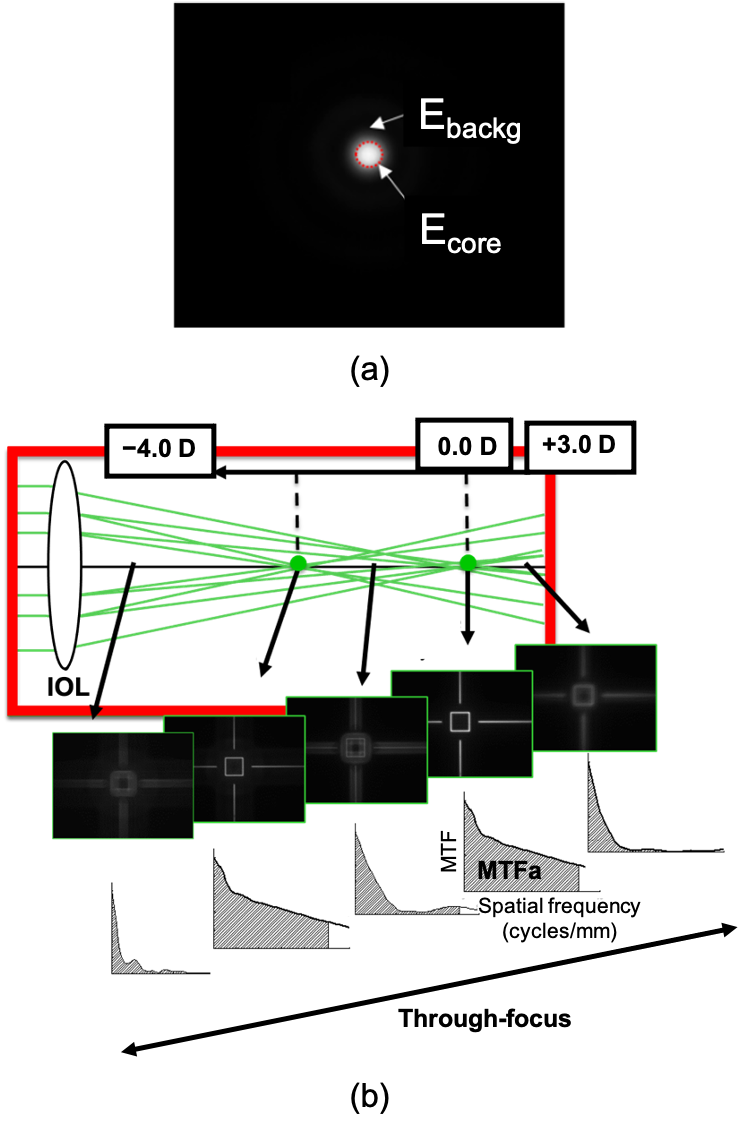


**Figure S4.** Images of the pinhole test formed by the model eye with an IOL immersed: **(a)** in linear grayscale of intensity, **(b)** intensity profile, and **(c)** in logarithmic grayscale.


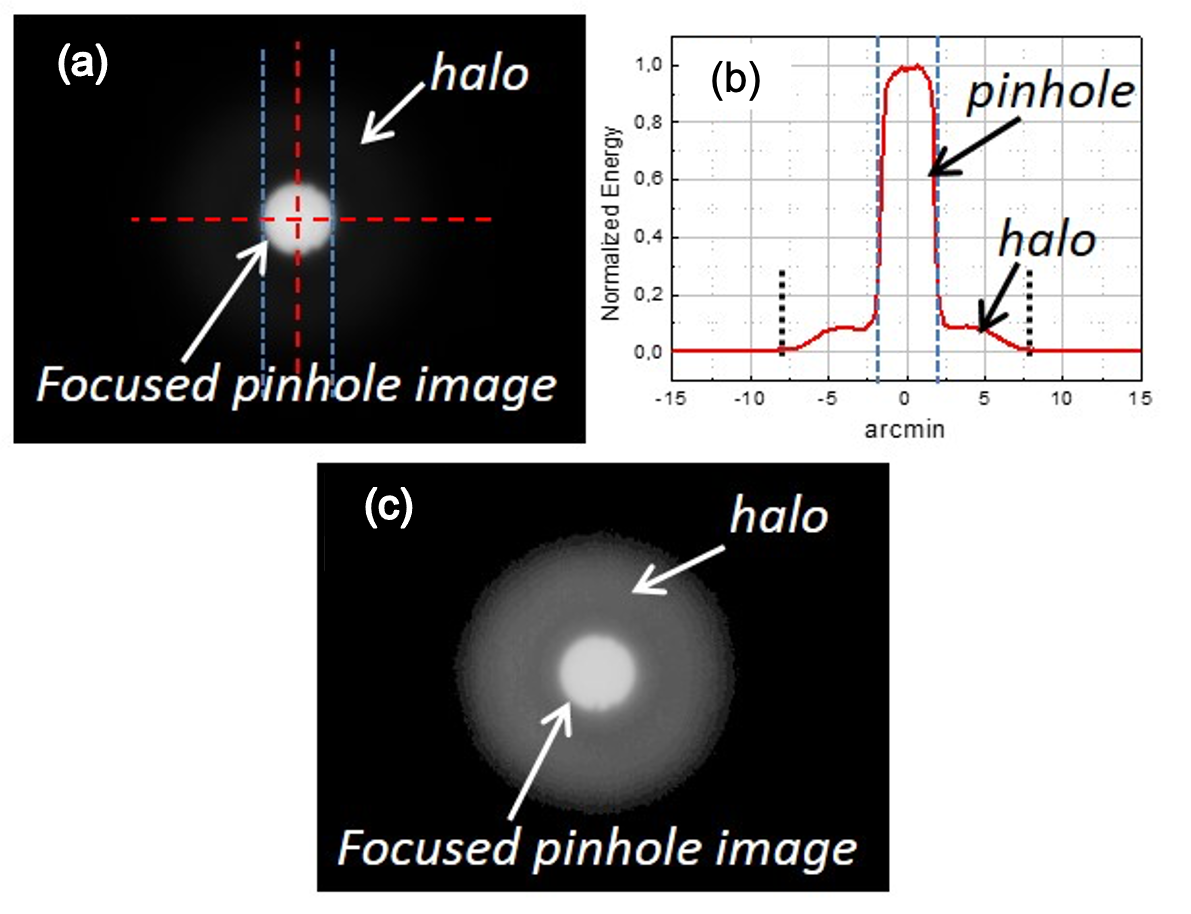


**Figure S5**. TF-EE and TF-MTFa measurements obtained in the laboratory experiment for the trifocal diffractive IOLs. **a** AT LISA tri; **b** FineVision under R, G, B lights and 4.5 mm pupil; **c** Polychromatic TF-EE and TF-MTFa curves of both IOLs. TF-EE, through-focus energy efficiency; TF-MTFa, through-focus area under the modulation transfer function; R, red; G, green; B, blue; IOL, intraocular lens


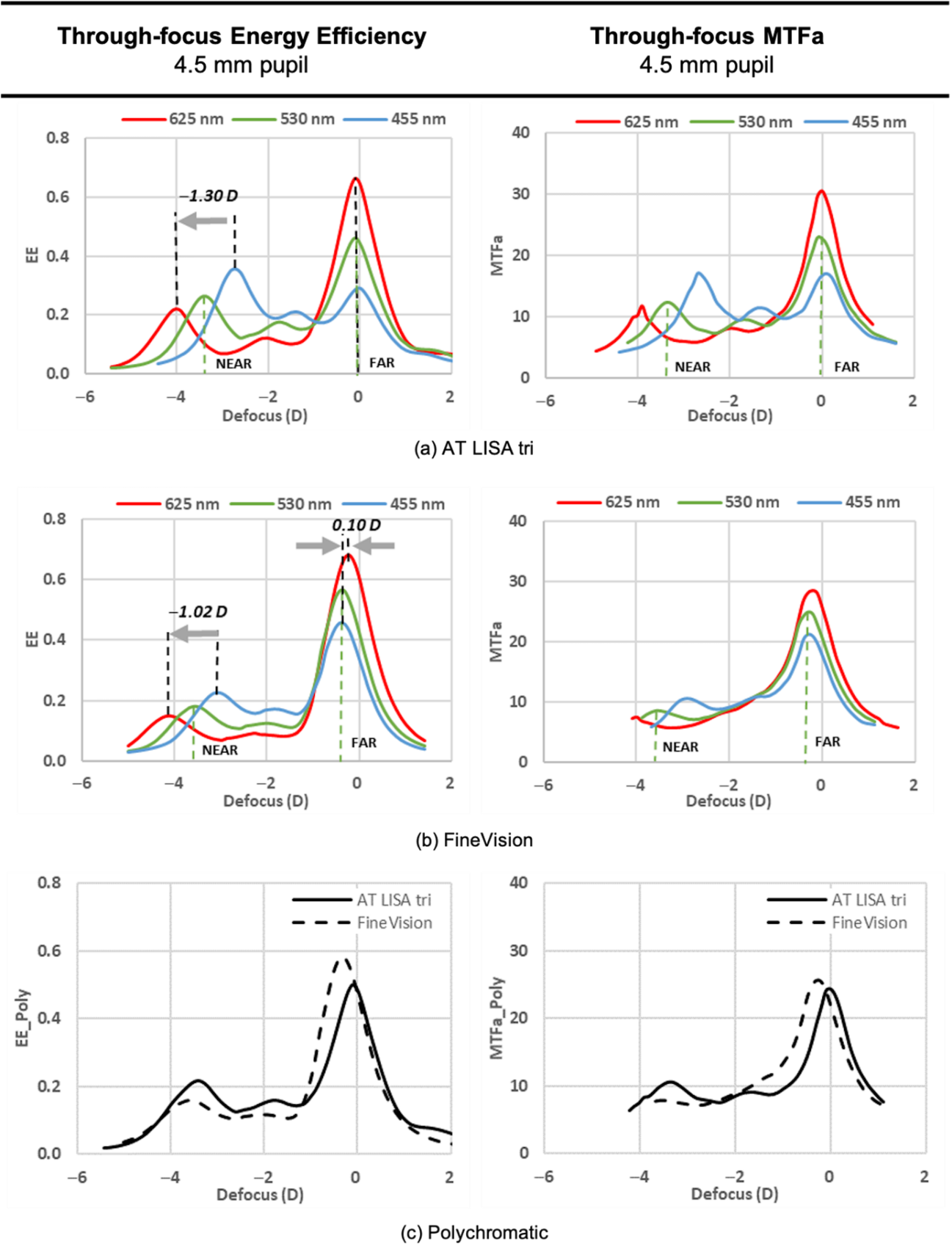


**Figure S6**: Far to near visual acuity (VA) differences under W, R, G, B lights for the subjects implanted with **(a)** AT LISA tri and **(b)** FineVision IOLs. Computed from Table 3. W, white; R, red; G, green; B, blue; IOL, intraocular lens


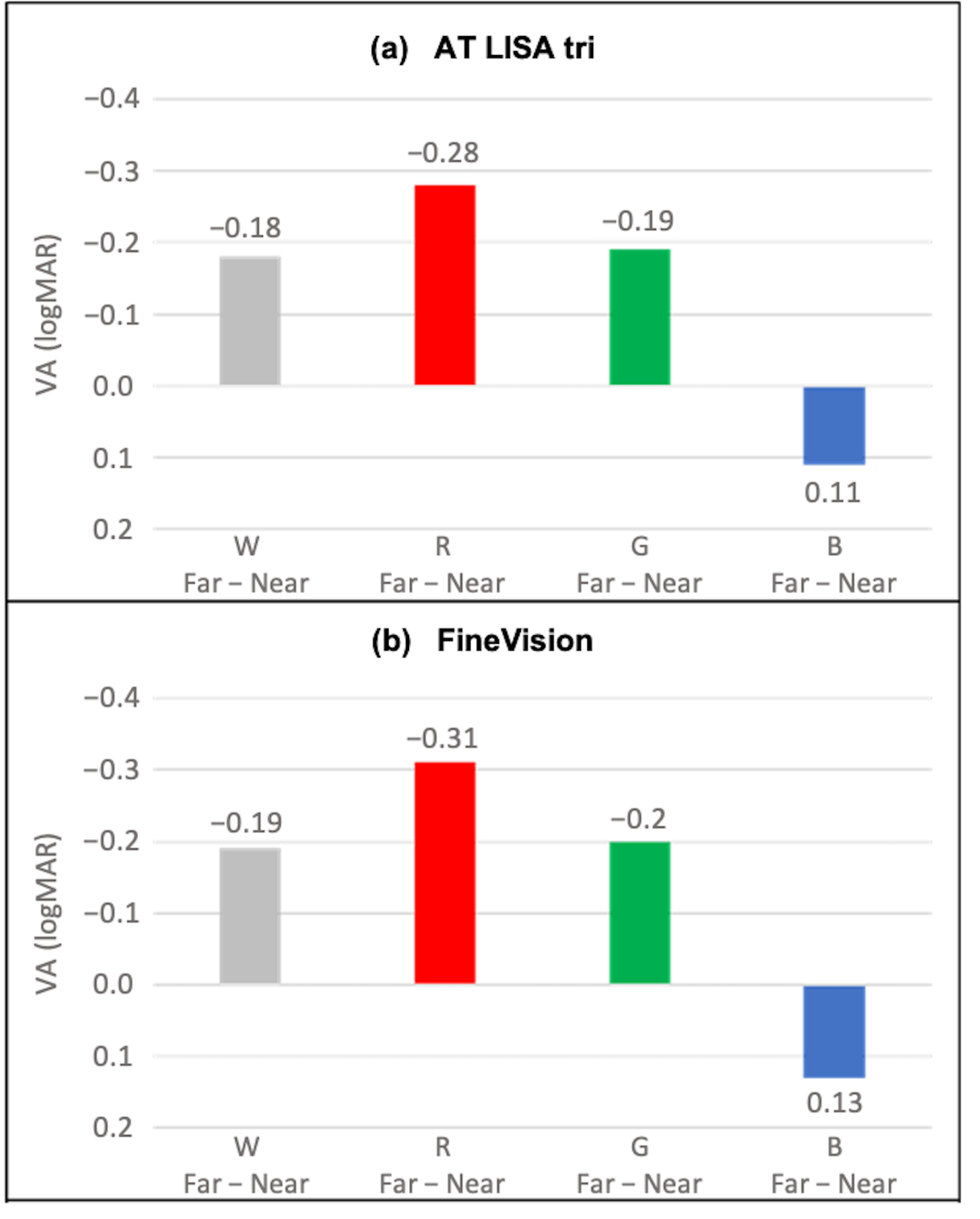

Supplement: Supplementary file 1 — Additional file 1: Table S1. Data of the light sources. Peak wavelength λ, full width at half maximum, correlated colour temperature, and chromatic coordinates in the CIE 1931 colour space chromaticity diagram. Table S2. Longitudinal chromatic aberration for the far and near IOL foci, obtained from the through-focus energy efficiency values of Figure S5. Figure S1. Schemes of the optical-bench testing setup. a Layout with an inset showing multiple foci; b Object tests; c Opto-mechanic depiction. Figure S2. Light sources. a to d Spectral band emissions of the LED sources: red, green, blue, and white with CCT 6500 K. e CIE 1931 colour space chromaticity diagram with the LED points. The straight lines are used to determine the weights of R, G, B mixture to be equivalent to W. Figure S3. Metrics for the optical characterization of IOLs in the laboratory experiment. a Energy efficiency calculated through the light-in-the-bucket ${E}_{core}/{E}_{total}$Ecore/Etotal ratio, with ${E}_{total}={E}_{core}+{E}_{backg}$Etotal=Ecore+Ebackg, applied to the image of the pinhole test. b Area under the MTF calculated from the line spread function. Scheme of the TF-MTFa measurement. Figure S4. Images of the pinhole test formed by the model eye with an IOL immersed: in linear grayscale of intensity, intensity profile, and in logarithmic grayscale. Figure S5. TF-EE and TF-MTFa measurements obtained in the laboratory experiment for the trifocal diffractive IOLs. a AT LISA tri; b FineVision, under R, G, B ligh [file 40662_2023_350_MOESM1_ESM.docx]
